# Modern Lipid Management: A Literature Review

**DOI:** 10.7759/cureus.9375

**Published:** 2020-07-24

**Authors:** Jahanzeb Malik, Hassan Shabeer, Uzma Ishaq, Humaira Chauhan, Hina Fatima Akhtar

**Affiliations:** 1 Cardiology, Rawalpindi Institute of Cardiology, Rawalpindi, PAK; 2 Hematology and Medical Oncology, Fauji Foundation Hospital, Rawalpindi, PAK; 3 Internal Medicine, KRL Hospital, Islamabad, PAK

**Keywords:** pcsk9 inhibitors, hyperlipidemia, atherosclerosis, coronary artery disease, hypercholesterolemia, ldl-c, alirocumab, evolocumab

## Abstract

Pro-protein convertase subtilisin/Kexin type 9 (PCSK9) inhibitors are relatively new, non-statin, lipid-lowering drugs that reduce low-density lipoprotein cholesterol (LDL-C) by 60%. PCSK9 inhibitors reduce the blood concentrations of cholesterol by the degradation of LDL receptors, which subsequently extracts cholesterol from cells. This leads to cardiovascular risk reduction in various at-risk populations, including atherosclerotic coronary artery disease. Despite their promise for advanced lipid-lowering ability, cost-effectiveness is a barrier to their routine use. While searching PubMed, we extracted land-mark trials on two of the anti-PCSK9 monoclonal antibodies, alirocumab and evolocumab. When combined with statins or ezetimibe, they show an exponential fall in LDL-C levels, helping achieve target values in high-risk populations and decreasing cardiovascular adverse events. Ongoing research is exploring the long-term efficacy of these antibodies in established coronary artery disease and familial hypercholesterolemia with more prospects for this novel lipid-lowering therapy.

## Introduction and background

For decades, statins have been the mainstay of treatment for hypercholesterolemia. They decrease cholesterol synthesis through the inhibition of 3-hydroxy-3-methylglutaryl-CoA (HMG-CoA) reductase, a rate-controlling enzyme in the mevalonate pathway [1]. Although they provide a successful reduction in the risk of major cardiovascular events and mortality for the at-risk population, there are patients for whom statins alone or in combination with multiple lipid-lowering agents are inadequate to reduce cardiovascular risk. Some patients have an intolerance to statins due to excessive symptoms and/or liver disease [2]. In those patients, other lipid-lowering agents like bile acid sequestrants or ezetimibe is normally insufficient [3]. 

In general cardiology practice, a large number of patients who are at high cardiovascular risk are below the target range for LCL-C levels. Recommended LDL-C should be below 70 mg/dl (<1.8 mmol/L) [4] and inadequate control of LDL-C shows a need for more effective treatments. It may also indicate non-compliance to treatment, especially in patients with multiple comorbidities who are taking a lot of medications, apart from the fact that 10% of the population is also intolerant to statins [5].

High levels of LDL-C are identified as genetic polymorphisms rather than lifestyle choices [6]. In the early 2000s, a genetic sequence was identified, which binds to LDL receptors, thus inhibiting the binding of LDL-C and its removal from the body. This, therefore, raises the concentration of LDL-C in the blood. This protein is named pro-protein convertase subtilisin/Kexin type 9 (PCSK9) [7]. The monoclonal antibodies that target PCSK9 for lowering plasma LDL-C concentration were approved in the European Society of Cardiology in their latest lipid guidelines [8]. Alirocumab and evolocumab are the two anti-PCSK9 monoclonal antibodies recently approved by the US Food and Drug Administration (FDA) for the treatment of high cardiovascular risk patients who do not adequately respond to basic lipid-lowering therapy [9-10]. Evolocumab is also approved for homozygous familial hypercholesterolemia and primary hypercholesterolemia [11].

Both alirocumab and evolocumab, in several landmark trials, have shown marked LDL-C reductions over 10 weeks (up to 70%). But this result comes at a high price tag. The cost is estimated to be $14000 per patient per year [12]. With only modest reductions in non-fatal atherosclerotic events, there has been strict allocation and regulations to restrict access to these therapies. 

This literature review aimed to assess the data available, evaluating the efficacy and safety of anti-PCSK9 monoclonal antibodies in patients with increased LDL-C levels.

## Review

Methods

For this literature review, randomized control trials were included, and to identify relevant studies, PubMed (2005 to 2020) was searched using “anti-PCSK9 antibodies”, “alirocumab”, and “evolocumab” as search terms. ClinicalTrials.gov was also searched for randomized control trials (RCTs).

For inclusion, all phase 3 trials that showed clear positive primary and secondary outcomes were included. Animal studies were excluded, as were case reports, opinions, editorials, and commentaries. Data on patient demographics and the extent of the disease, outcomes of the drug taken by the patients, and the safety of the patients were studied.

Results

**Figure 1:**
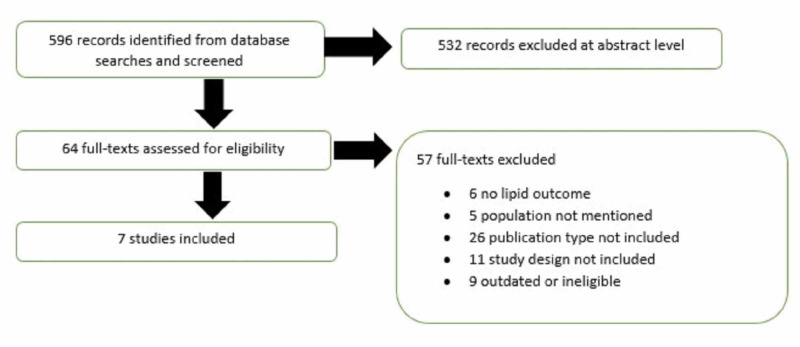
Results of the literature search

Seven articles were included in this review (Figure 1). The strength of evidence was found to be good. Several studies evaluated a PCSK9 inhibitor in varying cardiovascular risk patients, who failed to achieve target LDL-C levels with primary lipid-lowering strategies (LDL-C less than 70 mg/dL).

In patients at high risk for recurrent cardiovascular events, FOURIER (Further Cardiovascular Outcomes Research With PCSK9 Inhibition in Subjects With Elevated Risk; 2017) randomized 27,564 patients aged 40 to 85 years with clinically evident atherosclerotic cardiovascular disease [13-14]. This trial demonstrated that, relative to placebo, evolocumab significantly reduced LDL-C by 59% at 48 weeks (P=0.001). Relative to placebo, evolocumab reduced the risk of primary endpoint (1344 patients (9.8%) vs. 1563 patients (11.3%); hazard ratio, 0.85; 95% confidence interval (CI), 0.79 to 0.92; P<0.001) and the key secondary endpoint (816 (5.9%) vs. 1013 (7.4%); hazard ratio, 0.80; 95% CI, 0.73 to 0.88; P<0.001). There was no significant difference between the study groups concerning adverse events.

In another trial (ODYSSEY LONG TERM (Long-term Safety and Tolerability of Alirocumab in High Cardiovascular Risk Patients with Hypercholesterolemia Not Adequately Controlled with Their Lipid Modifying Therapy), 2015), in patients at high risk for cardiovascular events under the diagnosis of heterozygous familial hypercholesterolemia or established coronary artery disease, alirocumab reduced LDL-C levels by 62% in addition to high-intensity statin therapy with no significant adverse profile (P=0.001) [15]. It also lowered major adverse cardiovascular events as compared to placebo (1.7% vs. 3.3%; hazard ratio, 0.52; 95% confidence interval, 0.31 to 0.90; nominal P=0.02). In ODYSSEY COMBO II (Efficacy and Safety of Alirocumab Vs Ezetimibe on Top of Statins in High Cardiovascular Risk Patients with Hypercholesterolemia, 2015), alirocumab was used as an add-on therapy compared with ezetimibe in high-risk patients with inadequately controlled LDL-C levels [16]. At week 24, a 50% reduction in LDL-C was observed for alirocumab vs. 20% with ezetimibe. Alirocumab was well-tolerated, with no excess treatment-emergent adverse events.

In two open-label studies (OSLER (Open-Label Study of Long Term Evaluation Against LDL-C Trial) 1 and 2, 2019), evolocumab reduced LDL-C targets by 61% more than the standard care at 12 weeks [17-18]. In patients with high cardiovascular risk, the LAPLACE-2 (LDL-C Assessment with PCSK9 Monoclonal Antibody Inhibition Combined With Statin Therapy) study demonstrated that evolocumab, when added to atorvastatin, resulted in high rates of LDL-C targets at 12 weeks [19]. In statin-intolerant patients, two trials (GAUSS-1 and GAUSS-2 (Goal Achievement After Utilizing an Anti-PCSK9 Antibody in Statin Intolerant Subjects)) demonstrated that evolocumab led to higher reductions in LDL-C levels than placebo [20-21].

Discussion

Studies that were included in this literature review show that alirocumab and evolocumab are tolerated well and are effective in lowering LDL-C, which, in turn, lowers cardiovascular risk in not only the coronary artery disease subset but also in hypercholesterolemia or mixed lipidaemias. Both alirocumab and evolocumab reduced LDL-C targets in broad cardiovascular risk levels and comorbidities like diabetes, stroke, and chronic kidney disease.

Some studies have demonstrated a consistent lipid-lowering effect, irrespective of baseline LDL-C levels. For goal achievement in statin-intolerant patients, ODYSSEY ALTERNATIVE (Study of Alirocumab in Patients with Primary Hypercholesterolemia and Moderate, High, or Very High Cardiovascular Risk Who Are Intolerant to Statins) and GAUSS-2 failed to show promising lipid-lowering effect, but the data are still promising given the high baseline levels of LDL-C concentrations [21-22].

Rates of adverse events and of cardiovascular adverse events for both alirocumab and evolocumab were similar to those in the respective control arms of the studies. However, adverse events were lower, with alirocumab monotherapy as compared to a combination with statins. Although rare, treatment-emergent anti-bodies were reported in some studies [23-26]. It was reported at 12% in the ODYSSEY MONO study [27]. In evolocumab studies, treatment-emergent antibodies were reported in just one patient [28]. In familial hypercholesterolemia patients, the SPIRE (Studies of PCSK9 Inhibition and the Reduction of Vascular Events) trial demonstrated cardiovascular risk reduction with bococizumab [29].

As LDL-C is a recognized risk factor for cardiovascular disease, there is a significant reduction in cardiovascular events, but putting the study limitations into context, results should be interpreted cautiously [30]. Different methodologies were used in different trials and cardiovascular risk parameters were variably defined. To overcome this difference, analyzing the data according to the patient group is a good strategy. However, patient demographics also varied in different trials.

Study details and results are summarized in Table 1.

**Table 1:** Landmark trials LDL-C: Low-Density Lipoprotein Cholesterol; FOURIER: Further Cardiovascular Outcomes Research With PCSK9 Inhibition in Subjects With Elevated Risk; ODYSSEY LONG TERM: Long-term Safety and Tolerability of Alirocumab in High Cardiovascular Risk Patients With Hypercholesterolemia Not Adequately Controlled with Their Lipid Modifying Therapy; ODYSSEY COMBO II: Efficacy and Safety of Alirocumab Vs. Ezetimibe on Top of Statins in High Cardiovascular Risk Patients with Hypercholesterolemia; OSLER: Open-Label Study of Long-Term Evaluation Against LDL-C Trial; LAPLACE: LDL-C Assessment with PCSK9 Monoclonal Antibody Inhibition Combined With Statin Therapy; GAUSS: Goal Achievement After Utilizing an Anti-PCSK9 Antibody in Statin Intolerant Subjects; SPIRE: Studies of PCSK9 Inhibition and the Reduction of Vascular Events

Trial Name	Sample Size (n), End Point	PCSK9 Inhibitor Dose	Difference in LDL-C Change
FOURIER [13]	N=27,564 48 weeks	Evolocumab 140 mg once every two weeks or 420 mg once every one month	-59%, p=0.001
ODYSSEY LONG TERM [15]	N=812, 78 weeks	Alirocumab 150 mg once every two weeks	-62.6%, p=0.0842
ODYSSEY COMBO II [16]	N=720, 52 weeks	Alirocumab 75 mg once every two weeks	-50.6%, p=0.0001
OSLER [18]	N=1,255, 1 years	Evolocumab 420 mg once every one month	-56%, p=0.001
LAPLACE-2 [19]	N=2067, 12 week	Evolocumab 140 mg Q2W or 420 mg once every one month	-66 to 75%, p=0.001
GAUSS-1 [20, 21]	N=160, 12 week	Evolocumab 280 mg, 350 mg, 420 mg once every one month	-67%, p=0.001
c-1 [29]	N=17,000	Bococizumab 150 mg once every two weeks	-43% p=0.87

## Conclusions

This literature review provides an overview of all landmark trials for alirocumab and evolocumab, two anti-PCSK9 monoclonal antibodies approved by the FDA. Two large studies, named PROFICIO (Program to Reduce LDL-C and Cardiovascular Outcomes Following Inhibition of PCSK9 In Different Populations) and ODYSSEY, are further investigating these drugs. Using anti-PPCSK9 antibodies as an add-on therapy to statins or ezetimibe, it will allow more patients to achieve their LDL-C goal, with fewer adverse effects. In the statin-intolerant population, their drugs used as monotherapy can significantly reduce target LDL-C levels, conferring reduced cardiovascular risk.
